# A Vaccine Against Cervical Cancer: Context for the Global Public Health Practitioner

**DOI:** 10.9745/GHSP-D-18-00222

**Published:** 2018-12-27

**Authors:** Mary Carol Jennings, Anagha Loharikar

**Affiliations:** a International Vaccine Access Center, Department of International Health, Johns Hopkins Bloomberg School of Public Health, Baltimore, MD, USA.; b Vaccine Introduction Team, Global Immunization Division, U.S. Centers for Disease Control and Prevention, Atlanta, GA, USA.

## Abstract

Many low- and middle-income countries are moving to introduce HPV vaccine into their national immunization programs. To improve coverage, equity, and sustainability, public health officials and practitioners can use planning and implementation lessons learned, including successful school-based delivery strategies, innovative approaches to reach out-of-school girls, best practices for communication and social mobilization, and integration of services to reduce delivery cost. Policy makers, donors, and global partners should continue to consider ways to drive down costs of vaccine procurement.

## CERVICAL CANCER BURDEN AND HPV VACCINE RECOMMENDATIONS

As countries move to add primary prevention to their strategies to combat death and morbidity associated with cervical cancer, many practitioners in immunization as well as experts in non-communicable and communicable diseases will benefit from keeping up-to-date with recent developments in practice and implementation regarding human papillomavirus (HPV) vaccine delivery.

Persistent infection of cervical epithelial cells with “high-risk” carcinogenic types of HPV causes 99% of the estimated 530,000 global cases^1^ of cervical cancer that occur each year^2^^,^^3^—the majority of which occur in low- and lower-middle-income countries,^4^ where screening and treatment programs are not typically robust. HPV types 16 and 18 cause 70% of cancer globally; the contribution of 5 more high-risk HPV types accounts for 90% of the global cervical cancer burden.^5^

Three HPV vaccines are currently on the global market: a bivalent product (protecting against HPV types 16 and 18), a quadrivalent product (protecting against HPV types 6, 11, 16, 18), and a nonavalent product (protecting against HPV types 6, 11, 16, 18, 31, 33, 45, 52, 58).^4^ These vaccines are close to 100% efficacious at preventing HPV infection from the HPV types they target directly,^6^ with additional cross-protection against other HPV types.^7^^,^^8^ Multiple clinical trials, particularly of the bivalent and quadrivalent products (the first market entrants), have also demonstrated close to 100% efficacy in protecting against cervical intraepithelial neoplasia caused by HPV types covered by these vaccines.^9^^–^^12^ All 3 vaccines offer a similar, positive safety profile.^4^

The World Health Organization (WHO) recommends all countries include HPV vaccine in their national immunization schedule. WHO recommends 2 doses of HPV vaccine for girls ages 9–14 years, separated by a minimum interval of 6 months, and 3 doses of HPV vaccine for girls ages 15 years and over.^4^ Vaccination during pregnancy is not recommended^4^; however, accumulating safety evidence suggests no increased risk of adverse pregnancy outcomes.^13^ Immunocompromised youth, including anyone with HIV, should be vaccinated with 3 doses. Of note, neither HIV nor pregnancy testing are indicated as a prerequisite for receiving the vaccine.^4^ WHO recommends that, if feasible, countries vaccinate multiple age cohorts (e.g., 9–14 year-olds) in the first year of introduction. The existence of a cervical cancer screening or treatment program is not a prerequisite for vaccine introduction.^4^

We offer this commentary in the hope of focusing dialogue between and among public health practitioners and public health officials on key recent developments in the planning and implementation of HPV vaccination programs.

## SUPPORT FOR HPV VACCINE INTRODUCTION

Gavi, the Vaccine Alliance provides support for vaccine introduction and immunization programs in eligible countries; country eligibility for support from Gavi is chiefly determined by the gross national income (GNI) per capita, which determines the level of co-financing and nature of vaccine program support available.^14^ Between 2012 and 2016, both Gavi and vaccine manufacturers funded sub-national demonstration projects in countries around the world to better understand how to feasibly and sustainably deliver HPV vaccine to adolescents, a relatively new target age group that does not routinely access health services, especially in low- and middle-income countries. Gavi-eligible countries needed to demonstrate successful vaccination to this new target age group prior to requesting funds for national HPV vaccine introduction. Starting in 2017, Gavi shifted its focus from demonstration programs to national introductions, aiming to scale up early lessons learned and to accelerate progress toward the goal of protecting 40 million girls from cervical cancer by 2020 in Gavi-eligible countries.^15^ Following WHO recommendations, Gavi enacted policy to support vaccination of multiple age cohorts in the first year of program implementation for Gavi-eligible countries. However, due to current global vaccine shortage, some countries have been advised to target a single age group for the first year of introduction, with the potential to vaccinate multiple age cohorts in the future, as supply allows. Demonstrating experience in delivering the vaccine to adolescents is no longer a prerequisite for support of national implementation in these countries.

Following WHO's change in HPV vaccine policy and Gavi's shift in program support, many Gavi-eligible countries are moving rapidly to introduce HPV vaccine into their national immunization programs.

## PROGRAMMATIC CONSIDERATIONS FOR HPV VACCINE INTRODUCTION AND IMPLEMENTATION

Achieving high vaccination coverage through a routine immunization program among adolescent girls necessitates innovative delivery strategies and communication efforts.

Among 45 low- and middle-income countries surveyed in 2016 after having completed HPV vaccine demonstration programs or national introduction, most (87%) used primarily a school-based delivery strategy.^16^ While the majority (96%) of programs reporting data successfully achieved first-dose vaccination coverage of at least 70% among the target age group, only 83% of programs reporting data attained the same milestone for complete series coverage.^16^^,^^17^ The use of a school-based delivery strategy for other relevant vaccines has been successfully implemented in some countries^18^—for example, for second-dose measles vaccine at school entry^19^ and vaccines against tetanus, diphtheria, and pertussis.^20^^,^^21^ The use of school health programs to deliver other health services, such as vitamin A supplementation and deworming medications,^22^ is a well-established practice. However, while teachers can feasibly be trained to distribute tablets or medications, an injectable vaccine requires additional health worker involvement that can be disruptive or resource-intensive for national immunization programs to provide in the school setting.

Using a school-based vaccine delivery platform has effectively achieved high coverage for girls in school but poses an equity challenge for out-of-school youth, many of whom have poor access to health services and screening later in life.^23^ Despite the use of fixed-site and targeted outreach strategies to reach out-of-school girls in demonstration projects,^16^ few data-driven strategies to deliver HPV vaccines to out-of-school-girls have been designed and implemented, and fewer rigorously tested.^16^^,^^24^^,^^25^ Even in populations with high primary school enrollment, there may be poor school attendance among 9–14 year-olds. Unless social mobilization efforts are undertaken to ensure enrolled girls attend school on vaccination days, vaccination coverage will likely be low.^26^^,^^27^

School-based vaccine delivery models have effectively achieved high coverage for girls in school but pose an equity challenge for out-of-school youth.

To continue to build successful HPV vaccination programs, several types of stakeholders must be engaged in the program planning process. Regardless of how and where the vaccine is delivered, education stakeholders need to be involved in program planning and communication, as the adolescent age group is largely enrolled in primary school. Other key stakeholders include adolescent and youth service providers, community service organizations, local women's groups, family planning and reproductive health advocates, cervical cancer specialists, gynecology organizations, and HIV prevention and treatment groups. Vaccine delivery may also be a promising service for integration with other development or health services for girls, such as nutrition, economic empowerment, menstrual hygiene, and disease prevention, so stakeholders who are experts in those programs may be involved.

While at least 11 countries around the world, including Australia and the United States of America, routinely vaccinate boys with HPV vaccine, achieving high coverage among girls is a more cost-effective vaccination strategy in low- and middle-income countries than a “gender-neutral” vaccination strategy that immunizes both girls and boys.^4^ Countries can certainly choose to also vaccinate boys if this strategy is deemed financially and politically feasible; however, Gavi is currently only providing donor funding for vaccination of girls ages 9–14 years.

The current context of most countries focusing on vaccinating girls illuminates the importance of having a clear communication and social messaging campaign in place, with a realistic and nimble crisis communication strategy that can be activated quickly if rumors emerge.^16^ Vaccinating only girls can lead to rumors about the vaccine impacting fertility. Many countries have found that best practice is to have media, and well-trained media spokespersons, involved early in the planning, well ahead of vaccine introduction activities.

Although delivering vaccines to girls nationwide requires a different scale of resource commitment than a demonstration program, a number of potentially generalizable communication lessons can be drawn from studying programs that have implemented HPV vaccination to date.^16^ Program evaluations have shown how important it is for vaccination programs to be jointly “owned” by both the immunization program as well as educational institutions, for consent, social mobilization, logistics, and monitoring. Data from prior evaluations demonstrate that opt-out consent processes are generally acceptable and follow the consent format of other routine immunizations. Using an opt-in consent process can lead to rumors and misconceptions, but this may be mitigated by face-to-face communication with parents and communities.^27^ Experience responding to rumors and negative stories in the media has shown program implementers that social mobilization should happen well ahead of vaccine introduction.

Our understanding of best practices continues to evolve, highlighted by some best-case examples from Rwanda and Bhutan. In 2011, Rwanda became the first low-income country in the world to introduce HPV vaccine into its national program, and with strong leadership from its First Lady, partnership with industry, and effective, evidence-based mobilization efforts, has consistently reported between 93% and 96% full-course coverage.^28^^,^^29^ Bhutan, a lower-middle-income country and another early adopter, introduced HPV vaccine into its national immunization program in 2010, and with country ownership, a strong public-private partnership, an evidence-based and flexible delivery strategy, leadership from schools, and a proactive approach to media engagement, thereafter achieved consistent complete series coverage of over 90% among targeted 12-year-old girl cohorts, using a school-based delivery strategy.^17^

Although adolescence is arguably one of the healthiest periods of the life course, investment in this population, and inquiry into which services can be successfully and cost-effectively bundled with HPV vaccination, offers significant opportunity for impact.

## ECONOMIC CONSIDERATIONS FOR LOW- AND MIDDLE-INCOME COUNTRY INTRODUCTIONS

### Cost-Effectiveness

Overall, validated and relatively sophisticated economic models predict that HPV vaccination is very cost-effective in most countries, particularly in low-income countries.^30^ Introducing an expensive new vaccine constitutes a significant investment on behalf of a government, with vaccine cost accounting for approximately half of the total cost of procurement and delivery.^31^ Delivery costs reported across demonstration programs and delivery strategies ranged from US$1.11 to $9.21 per dose.^27^ Bhutan spent US$2.40 to deliver each HPV vaccine dose in a well-documented 2010 evaluation of its national program.^17^ In Tanzania, a 2012 analysis estimated a delivery cost using a periodic school-based campaign delivery strategy of US$3.09 per dose; this cost estimate was in addition to the cost of vaccine,^32^ and the program was categorized as a very cost-effective intervention.^33^

Robust economic models predict that HPV vaccination is very cost-effective in most countries, particularly in low-income countries.

### Resources to Support New Vaccines for Low- and Middle-Income Countries

All 3 HPV vaccine products on the global market are currently WHO-prequalified; as of August 2018, the quadrivalent and bivalent products are approved for Gavi funding support to eligible countries. Gavi provides a vaccine introduction grant as part of its initial start-up package to a country to cover operational costs and social mobilization efforts. Gavi-eligible countries can also procure the prequalified HPV vaccines for US$4.60 per dose (bivalent product) and US$4.50 per dose (quadrivalent product).^15^ However, as country economic indicators (i.e., GNI) improve to the point that they are no longer eligible for Gavi funding, countries must budget an incrementally larger share of the costs each year until they entirely self-fund both vaccine procurement and delivery costs. For countries whose economic indicators (i.e., GNI) improve to the point that they are no longer eligible for Gavi funding, as well as for middle-income countries that were never Gavi-eligible, these recurring programmatic and procurement costs represent a significant portion of national immunization budgets. Depending upon the vaccine, manufacturers may agree to continue offering Gavi-negotiated prices to countries for a selected number of years after transition. However, we note the critical need for donor mechanisms to ensure that middle-income countries can introduce HPV vaccines, and that transitioning countries can sustain new introduction decisions.

### Innovations and Potential Shifts in Cost

Looking forward, new developments may be able to reduce HPV vaccine procurement and delivery costs. The eventual market entry of vaccines manufactured by companies based in low- and middle-income countries and owned by local entities may create the same downward pressures on prices as we have seen with multiple other medicines and biologics.^34^^,^^35^ One of the key barriers to development of such low-cost second-generation HPV vaccines is the lack of standardized and widely accessible laboratory serology tests and assays to assess how new vaccines perform against the currently licensed vaccines. An initiative intended to standardize and evaluate new laboratory tests—developed by a variety of institutions—to address this gap was established at the beginning of 2017 at the U.S. National Cancer Institute.^36^

Other factors may also play a role in reducing expected costs of program implementation. For example, an analysis by Gavi and WHO anticipates that national programs will harness economies of scale much more effectively than small demonstration programs were able to do.^31^

National HPV vaccination programs will likely harness economies of scale much more effectively than small demonstration programs.

Data on whether a 1-dose schedule confers adequate levels of protection show promise,^37^^–^^40^ but the science available does not yet provide definitive guidance for policy.^4^^,^^41^^,^^42^ The U.S. National Cancer Institute is currently conducting a large randomized controlled trial to evaluate the efficacy of a single-dose regimen in Costa Rica, with availability of results targeted for 2023.^42^^,^^43^

### Relationship to Cervical Cancer Screening and Treatment

As countries introduce and scale up HPV vaccination programs, cervical cancer screening remains important for women who do not get vaccinated as children and for women who may have been infected with a high-risk HPV type that is not included in the vaccine. As national stakeholders in cancer and chronic diseases come together with immunization programs and their advisory bodies to make policy on HPV vaccination, they have an important opportunity to also inform their national policies on cervical cancer screening and surveillance programs.

## Notes

**Peer Reviewed**

**Funding**: None.

**First Published Online:** November 20, 2018
